# Differences in menopausal quality of life, body appreciation, and body dissatisfaction between women at high and low risk of an eating disorder

**DOI:** 10.1002/brb3.3609

**Published:** 2024-07-15

**Authors:** Sophie Temple, Eef Hogervorst, Gemma L. Witcomb

**Affiliations:** ^1^ School of Sport, Exercise, and Health Sciences Loughborough University Loughborough UK

**Keywords:** body image, disordered eating, menopause, quality of life

## Abstract

**Objective:**

Experiences of menopause and quality of life during menopause can vary extensively among women. While menopause has been associated with negative impacts on eating and body image, it is unclear to what extent quality of life differs by eating disorder risk status. The aim of this study was to explore how menopause symptoms and quality of life differ between those women at high‐ or low‐risk of an eating disorder and the potential protective role of body appreciation.

**Method:**

This cross‐sectional survey study explored differences in menopausal quality of life, body appreciation, and body dissatisfaction among women classified as high‐ or low‐risk of an eating disorder as part of a wider survey on aging, health, and psychological complaints during midlife. Participants were 255 females aged between 40 and 60 years. Participants were classified as high‐risk and low‐risk of an eating disorder based on Eating Attitudes Test‐26 (EAT‐26) scores. Differences between groups on the Menopause‐Specific Quality of Life Questionnaire (MENQOL), Body Shape Questionnaire (BSQ‐16), and Body Appreciation Scale–2 were analyzed. The predictive relationship between menopausal quality of life and body appreciation was also explored.

**Results:**

Participants in the high‐risk group (*n* = 111) reported significantly poorer menopausal quality of life compared to the low‐risk group (*n* = 144), scoring significantly higher on the sexual, physical, and psychosocial subscales of the MENQOL. The high‐risk group also had significantly greater body dissatisfaction and less body appreciation than the low‐risk group. Overall, menopausal quality of life was a significant predictor of body appreciation.

**Discussion:**

Women with greater eating disorder risk may be faring less well with menopause. Treating and preventing menopause‐related eating disorders will benefit from interventions aimed at not only reducing body dissatisfaction, but actively bolstering body appreciation and supporting the sexual, physical, and psychosocial aspects of the menopausal transition.

## Graphical abstract

This study suggests that women who experience eating pathology at midlife age may experience a lower quality of life during the menopausal transition. The study concludes that fostering greater body appreciation, and supporting the sexual, physical and psychosocial aspects of the menopausal transition may help reduce eating disorder risk during the menopausal transition.

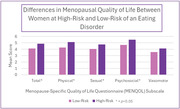

## 1 INTRODUCTION

Just as puberty plays a significant role in the development of eating disorders during adolescence and early adulthood, (Mangweth‐Matzek et al., 2023) the menopausal transition could similarly be a risk factor for the development of eating disorders during midlife (Mangweth‐Matzek et al., 2021). Many studies show that eating disorders occur in people of all ages and genders and are not limited solely to young women (Mangweth‐Matzek et al., 2023). While research on the association between eating disorder pathology and the menopausal transition is in its infancy, recent studies have highlighted the predictive value of menopausal symptomatology when assessing women at midlife age for eating and body image pathology (Mangweth‐Matzek et al., 2021).

The menopausal transition is referred to as the gradual transition, over a number of years, to the end of menstruation, due to the cessation of ovarian reproductive function. This transition encompasses endocrinal changes in the hormones estrogen, progesterone, and testosterone, which can induce an array of vasomotor, psychological, sexual, and physical changes, which can influence women's quality of life (Gatenby & Simpson, 2024). Although the menopausal transition can have a negative influence on women's body image, through changes in weight and body composition sometimes inducing body dissatisfaction and reduced self‐esteem, research also supports the existence of menopausal‐induced positive influences on body image. Entering the menopausal transition can often induce a sense of freedom from appearance‐based concerns alongside a reduction in self‐objectification and body monitoring, as individuals gain more meaningful life experiences (Rubinstein & Foster, 2013).

Early research suggests that eating disorder vulnerability may reach a temporary peak during the perimenopausal phase of the menopausal transition (Tepper et al., 2012). Differing trajectories of estrogen change have been observed during the menopausal transition, with some women experiencing a slower, more gradual estrogen decline whereas others experience more volatile highs and lows (Tepper et al., 2012). Although women may be categorized within the same menopausal stage based on their menstrual patterns, there can still be significant variations in the trajectories of their reproductive hormone levels, and, as such, effects on the body associated with menopause such as changes in weight, body composition and body shape (Fenton, 2021). This insight may help explain the absence of consistent and significant differences observed in the body image perceptions of women at different stages—premenopausal, perimenopausal, and postmenopausal. Research indicates that menopausal changes, driven by variations in hormones, play a crucial role in influencing body image disturbances during the menopausal transition (Pearce et al., 2014; Rubinstein & Foster, 2013). Perhaps if women experience a more sudden and erratic trajectory of hormonal changes, this could impact their body image more severely as they have less time to adjust to the onset of changes in their physical appearance, which may stray from Western beauty ideals.

Consequently, Baker and Runfola (2016) hypothesized that women are most vulnerable to developing eating disorder pathology during the perimenopausal phase when estrogen variability is typically the most volatile. This “perimenopausal vulnerability” hypothesis was supported by Mangweth‐Matzek et al. (2014), who found that perimenopausal women reported a significantly greater prevalence of eating disorders, higher self‐ratings of “feeling fat,” and greater body dissatisfaction than premenopausal women (Mangweth‐Matzek et al., 2014). Similarly, Khalil et al. found perimenopause to be associated with more binge eating than the premenopausal and postmenopausal stages (Khalil et al., 2022). However, other studies investigating overall eating pathology (Mangweth‐Matzek et al., 2021; Thompson & Bardone‐Cone, 2019) have found no significant differences in measures of eating pathology and body image between premenopausal, perimenopausal, and postmenopausal women. This may be attributed to the overlap of symptoms across perimenopausal and postmenopausal phases, which render the objective classification of menopausal status challenging for both researchers and for women themselves (Sherman, 2005).

More recent research acknowledges that while eating pathology often manifests when menopausal symptoms are most severe, this does not necessarily coincide with the perimenopausal phase for all women. Mangweth‐Matzek *et al.* (2021) found no significant differences in the prevalence of eating pathology between women compared by menopausal stage groups; however, differences were found between women reporting severe menopausal symptomatology compared to those with low symptomatology, assessed by the menopausal rating scale (MRS) (Mangweth‐Matzek et al., 2021). This suggests that adopting measures of assessing menopausal symptomatology, such as the Menopause‐Specific Quality of Life Questionnaire (MENQOL) (Hilditch et al., 1996) may be more reliable indicators of the impact of menopause than age or menstrual status.

The MENQOL (Hilditch et al., 1996) incorporates the presence of the physical, psychosocial, sexual, and vasomotor symptoms of the menopausal transition and, critically, the subjective evaluation of the distress that individuals attribute to each of these symptoms. Poor menopausal quality of life may induce and exacerbate eating pathology through several mechanisms. For example, research using non‐menopausal populations suggests that when episodes of binge eating are prompted by negative emotions, a negative reinforcement pattern often occurs. This is hypothesized to occur because the release of dopamine and serotonin during binge episodes can lead to a reduction in negative emotions, reinforcing bingeing behavior (Anaya et al., 2023; Naef et al., 2015). Furthermore, sustained periods of low mood can prompt certain individuals to resort to food restriction as a coping mechanism, often driven by various motives such as seeking a sense of control over their emotions and circumstances (Thompson et al., 2022). It is plausible that a diminished menopausal quality of life, arising from the manifestation of severe menopausal symptomology, could likewise contribute to the development of eating disorder pathology through these mechanisms. However, while menopausal symptomology has been established as a predictor of eating pathology (Mangweth‐Matzek et al., 2021), the extent to which different symptoms (physical, psychosocial, vasomotor, or sexual) may be more or less influential is unclear.

Eating pathology is widely acknowledged to be linked with body dissatisfaction in non‐menopausal populations. For some women, entering the menopausal transition can signify a transition into a new life stage. Unfortunately, in Western society, aging—particularly for women—is still associated with a number of significant negative stereotypes, such as becoming less valued or capable, both physically and socially. Women's abilities are also often underestimated or disregarded (Ng, 2021). Such stereotypes can have a negative impact on an individual's sense of self‐worth and body satisfaction. Indeed, research has shown that many women in their 50s engage in extreme dieting and restrictive eating behaviors to delay the onset of natural signs of aging and may avoid social situations due to poor body esteem (McLaren & Kuh, 2004). Recent longitudinal research has also revealed a significant association between higher body dissatisfaction and greater negative affect, psychosocial impairment, reduced enjoyment of physical activity, and poorer psychological, social, and physical quality of life. This highlights the significant impact of body image disturbance on multiple dimensions of women's lives across their lifespan (Kilpela et al., 2023). Overall, it appears that body dissatisfaction is prevalent in mid‐life women and that aging does not protect women from weight preoccupation and appearance‐based concerns. As such, the menopausal transition is likely to be playing a role.

Research across many countries consistently supports the association between menopausal symptoms and changes in body image. Mangweth‐Matzek et al. found that higher menopausal symptom severity, as measured via the MRS, was associated with lower ratings of body, weight, and shape satisfaction using modified questions from the E‐D Eating Disorder Examination Questionnaire (Mangweth‐Matzek et al., 2021). Similarly, Nazarpour et al, (2021) found negative associations between somatic, psychological, and urogenital symptoms and four dimensions of body image (head and face, upper limbs, lower limbs, and overall) (Nazarpour et al., 2021). In other research, appearance evaluation was significantly associated with the frequency of vasomotor and psychological symptoms (Sakson‐Obada & Wycisk, 2015). The more frequently women experienced these symptoms, the lower their estimations of their physical attractiveness.

It is likely that the positive facets of body image may reduce menopausal eating disorder risk (Quittkat et al., 2019). For example, body appreciation incorporates accepting and holding favorable attitudes toward the body regardless of physical appearance, weight, shape, and imperfections. Body appreciation may prevent the development of eating pathology despite (perceived) weight gain and/or changes in body composition by helping women to dismiss unrealistic body standards portrayed by the media, reducing their consumption of appearance‐focused media, and reducing their tendency to self‐objectify (Koller et al., 2020; Linardon et al., 2022). Although the relationship between body appreciation and eating pathology has not been studied specifically in menopausal women, studies support that the menopausal transition is a period in women's lives where body image perceptions are vulnerable (McLaren & Kuh, 2004; Ng, 2021). Therefore, further investigation into the protective potential of this construct against menopausal eating disorders is warranted.

The present study sought to ascertain whether women with a high‐risk of an eating disorder exhibit more severe menopausal symptoms (sexual, physical, psychosocial, and vasomotor) and are more bothered by these symptoms, and thus display a reduced menopausal quality of life than women with low‐risk, and whether the two groups differ in body dissatisfaction and body appreciation. Furthermore, secondary analysis sought to explore whether menopausal quality of life is predictive of body appreciation, rather than body dissatisfaction, to expand existing research exploring the relationship between menopausal symptomology and body image. The core objective of the present study was to ascertain whether there is a significant difference in the menopausal quality of life, body appreciation, and body dissatisfaction between women who are at high risk of an eating disorder compared to women who are at low risk of an eating disorder. The overall aim was to provide insights into the characteristics of individuals who are vulnerable to developing an eating disorder during midlife. Given that previous research supports a positive association between menopausal symptoms and eating pathology and the long‐established associations between eating disorders and body image pathology, the present study hypothesized that the high‐risk eating disorder group would report a significantly poorer menopausal quality of life, significantly higher body dissatisfaction, and significantly lower body appreciation compared to the low‐risk group. It was also hypothesized that menopausal quality of life would be a significant predictor of body appreciation, with poorer quality of life predicting reduced levels of body appreciation.

## 2 METHODS

A cross‐sectional design was used, utilizing an anonymous online survey, hosted in Online Surveys, to collect data relating to eating disorder pathology, body dissatisfaction, body appreciation, and menopausal quality of life. The study was advertised between November 2022 and February 2023, using virtual posters (on Facebook, Twitter, and LinkedIn) and physical posters (in social spaces around a UK town center and university campus). Interested participants could access the study information, consent form, and the survey via a QR code or URL on the poster.

### 2.1 Participants

An opportunity sample of 286 women aged between 40 and 60 years completed the survey. After excluding responses from ineligible participants (e.g., assigned male at birth), responses with missing data, and those whose responses were insufficient to permit classification into eating disorder categories (see below), a total of 255 participants were included. All respondents gave informed consent, and the study was approved by the Ethics Committee of Loughborough University (Review Reference: 2023‐13264‐12696).

### 2.2 Study instruments

#### 2.2.1 Demographic questions

Demographic data including weight and height, marital and employment status, birth year, ethnicity, number of children, and identification of any notable caregiving responsibilities was collected via self‐report in the online survey.

#### 2.2.2 Eating attitude test (Garner et al., 1982)

The Eating Attitudes Test‐26 (EAT‐26) questionnaire was used to assess eating disorder risk. Participants rated the frequency, from “Always” to “Never,” with which they engage in 26 eating cognitions/behaviors. For example, “I am terrified about being overweight” and “I find myself preoccupied with food.” The 26 items form three subscales: (1) dieting, (2) bulimia and food preoccupations, and (3) oral control. Participants were grouped as either “high‐risk” or “low‐risk” according to their overall scores on the EAT‐26, calculated by summing their scores from each subscale. High scores denote an increased risk of an eating disorder due to concerns regarding body weight, shape, and eating. A cut‐off score of greater than or equal to 11 for “high‐risk” was used, previously demonstrated to improve sensibility and sensitivity rates in individuals with bulimia nervosa, binge eating disorder, and eating disorders not otherwise specified (Orbitello et al., 2006). Cronbach's alpha was .87, indicating good internal consistency.

#### 2.2.3 Body shape questionnaire (BSQ‐16) (Avalos et al., 2005)

To assess body shape dissatisfaction and preoccupation typically associated with eating disorders (Slevec & Tiggemann, 2011), the 16‐item version of the BSQ‐16 (Cooper et al., 1987) was administered. Questions assess pertinent domains of body image, including preoccupation with weight/shape (e.g., “Have you noticed the shape of other women and thought your own shape compared unfavorably”), embarrassment (e.g., “Have you not been out to social occasions because you have felt bad about your shape”), self‐consciousness (e.g., “Have you avoided situations where people may see your body, e.g., communal changing rooms or swimming baths”), and excessive feelings of fatness after eating (e.g., “Has feeling full (e.g., after a large meal) made you feel fat?”). Item 7—“Have you imagined cutting off the fleshy areas of your body?”—was removed as it was considered potentially distressing by the ethics committee. Responses are scored on a 6‐point scale from “Never” (1) to “Always” (6). Higher summed scores denote greater concern/preoccupation with body shape and weight. Cronbach's alpha was .97, indicating excellent internal consistency.

#### 2.2.4 Body appreciation scale–2 (BAS‐2) (Avalos et al., 2005)

The BAS‐2 (Avalos et al., 2005) is a measure of positive body image that assesses acceptance and favorable opinions towards the body (e.g., “I feel that my body has at least some good qualities” and “I appreciate the different and unique characteristics of my body”) over 10 questions, each scored using a 5‐point Likert scale from 1 (‘Never’) to 5 (‘Always’) with higher summed scores indicating greater body appreciation. Cronbach's alpha was .96, indicating excellent internal consistency.

#### 2.2.5 Menopause‐Specific Quality of Life Questionnaire (MENQOL) (Hilditch et al., 1996)

The MENQOL (Hilditch et al., 1996) was used to assess menopause‐specific health‐related quality of life over the last month. Each of the 29 items assesses the impact of one of four domains of menopausal symptoms: vasomotor (e.g., “hot flashes” and “night sweats”), psychosocial (e.g., “Being dissatisfied with my personal life”), physical (e.g., “Weight gain”), and sexual symptoms (e.g., “Vaginal dryness during intercourse”). Symptoms are rated as being present or not, and if present, rated on a 7‐point scale from “not bothersome” (0) to “extremely bothersome” (6). Means are computed for each subscale, and subscale means are summed to give an overall total. Higher scores indicate poorer menopausal health‐related quality of life.

### 2.3 Statistical analysis

Statistical analyses were conducted using IBM SPSS V.28.0 (IBM) with statistical significance set as *p* < .05. Variables were checked for normality using Kolomogrov–Smirnov tests and histograms. Pairwise comparisons were conducted using gamma generalized linear models (GzLM), an extension of the general linear model, that does not require data normality or homoscedasticity (Ng & Cribbie, 2017), to examine the differences on MENQOL total score and the vasomotor, psychosocial, physical, and sexual subscales, BSQ‐16, and BAS‐2 scores between the “high‐ risk” and “low‐ risk” ED group.

Gamma distributions with log link for positively skewed variables were used to account for non‐normally distributed data. However, GzLM analysis cannot handle negative or null values (Ng & Cribbie, 2017) and subsequently removes data with null or negative change scores in analyses. Therefore, where participants had an EAT‐26 and/or MENQOL total/subscale score of zero, these were transformed to positive values by adding (+1) as a constant factor to each respective outcome. Given the potential confounding influence of body mass index (BMI) and age on body image, body appreciation, and menopausal quality of life outcomes, all GzLMswere adjusted by including age and BMI as covariates.

Secondary analyses were conducted using a simple linear regression to investigate the predictive relationship between menopausal quality of life and body appreciation. The assumptions of linearity, normality, homoscedasticity, and independence were assessed and met. The results were interpreted considering the coefficient of determination (R‐squared), standardized beta coefficients, and significance levels. Age and BMI were controlled for as covariates to ensure the specificity of the relationship between MENQOL and BAS‐2 scores.

An a priori power analysis was conducted using G*Power version 3.1.9.7 (Faul et al., 2007) to determine the minimum sample size required to test whether menopausal quality of life was a significant predictor of body appreciation. Results indicated the required sample size to achieve 80% power for detecting a medium effect, at a significance criterion of α = .05, was *N* = 89 for simple linear regression. Thus, the obtained sample size of *N *= 255 was adequate to test the study hypothesis.

## 3 RESULTS

### 3.1 Participants

Using scoring guidelines from the EAT‐26 (Garner et al., 1982), 111 participants were categorized as “high risk” for an eating disorder (score > 11) and 144 were categorized as “low‐risk” (score < 11). The demographic characteristics of participants were as follows: 90% identified as White, 78% reported having one or more child, 76% were classified as married or in a partnership, and 19% reported significant caring responsibilities. The mean age of the high‐risk group was 50.41 years (SD = 4.74), while the mean age of the low‐risk group was 51.87 years (SD = 4.93). A significant difference in age was found between the two groups, *t* [1, 255] = 2.389, *p* = .018). The BMI of the high‐risk group was 29.20 (SD = 6.62), whereas the BMI of the low‐risk group was 26.69 (SD = 6.57). A significant difference in BMI was observed between the two groups, *t* [1, 255] = −3.01, *p* = .003).

### 3.2 Eating disorder risk, menopausal quality of life, and body image

The results of the GzLM indicate that the high‐risk ED group scored significantly higher (*M* = 4.86, SD = 1.42) than the low‐risk ED group (*M* = 4.14, SD = 1.33) on the MENQOL total score. They also scored significantly higher on the sexual (*M* = 4.74, SD = 2.24), physical (*M* = 5.11, SD = 1.47), and psychosocial (*M* = 5.50*, SD* = 1.82) subscales, compared to the low‐risk group (*M*, SD—4.06 (2.14), 4.25 (1.39), and 4.67 (1.71), respectively). No significant difference was found between high‐risk (*M* = 4.11, SD = 4.16) and low‐risk (*M* = 3.55, SD = 1.86) groups on the vasomotor subscale of the MENQOL. There was also a significant difference in body dissatisfaction and body appreciation between groups, with the high‐risk group scoring significantly higher on the BSQ‐16 (*M* = 63.91, SD = 17.62) than the low‐risk group (*M* = 35.79*, SD *= 15.72), and significantly lower (*M* = 24.40*, SD* = 8.78) on the BAS‐2 than the low‐risk group (*M* = 33.10, SD = 7.99). See Table 1.

**TABLE 1:** Differences in BSQ‐Menopause‐Specific Quality of Life Questionnaire (MENQOL total score and subscales), body appreciation (BAS‐2) and body dissatisfaction (BSQ‐16) score according to eating disorder risk group ,

	Mean (SD)		*F*	*p* ^a^
	High risk (*N =* 111)	Low risk (*N = *144)		
MENQOL—Total	4.86 (1.42)	4.14 (1.33)	12.53	**<.001**
MENQOL—Vasomotor	4.11 (4.16)	3.55 (1.86)	3.15	.076
MENQOL—Psychosocial	5.50 (1.82)	4.67 (1.71)	7.42	**.006**
MENQOL—Sexual	4.74 (2.24)	4.06 (2.14)	6.12	**.013**
MENQOL—Physical	5.11 (1.47)	4.25 (1.39)	15.65	**<.001**
BAS‐2	24.40 (8.78)	33.10 (7.99)	55.21	**<.001**
BSQ‐16	63.91 (17.62)	35.79 (15.72)	108.75	**.000**

^a^ Adjusted for the covariates age and body mass index.

Abbreviations: MENQOL, Menopause‐Specific Quality of Life Questionnaire; BAS‐2, Body Appreciation Scale–2; BSQ‐16, Body Shape Questionnaire‐16.

### 3.3 Menopausal quality of life and body appreciation

Table 2 shows the bivariate correlations between all of the variables. All were significant at *p *< .002, except for EAT‐26 and MENQOL vasomotor (*p* = .052) and MENQOL social (*p* = .087).

**TABLE 2:** Bivariate correlations between Menopause‐Specific Quality of Life Questionnaire (MENQOL total score and subscales), eating disorder symptomology (EAT‐26), body appreciation (BAS‐2), and body dissatisfaction score (BSQ‐16).

	EAT‐26	BAS‐2	BSQ‐16	MENQOL—Total	MENQOL—Vasomotor	MENQOL—Psychological	MENQOL—Physical	MENQOL—Sexual
EAT‐26		−.468^**^	.654^**^	.222^**^	.122	.216^**^	.263^**^	.107
BAS‐2	−.468^**^		−.703^**^	−.421^**^	−.289^**^	−.384^**^	−.382^**^	−.249^**^
BSQ‐16	.654^**^	−.703^**^		.464^**^	.330^**^	.388^**^	.525^**^	.223^**^
MENQOL—Total	.222^**^	−.421^**^	.464^**^		.734^**^	.789^**^	.808^**^	.716^**^
MENQOL—Vasomotor	.122	−.289^**^	.330^**^	.734^**^		.423^**^	.518^**^	.282^**^
MENQOL—Psychosocial	.216^**^	−.384^**^	.388^**^	.789^**^	.423^**^		.652^**^	.387^**^
MENQOL—Physical	.263^**^	−.382^**^	.525^**^	.808^**^	.518^**^	.652^**^		.399^**^
MENQOL—Sexual	.107	−.249^**^	.223^**^	.716^**^.	.282^**^	.387^**^	.399^**^	

*Note*: *N* = 255.

** Correlation is significant at the 0.01 level (2‐tailed).

Abbreviations: EAT‐26, Eating Attitudes Test‐26; BAS‐2, Body Appreciation Scale–2; BSQ‐16, Body Shape Questionnaire‐16.

Simple linear regression was used to test if menopausal quality of life significantly predicted body appreciation. The overall regression was statistically significant (*R^2^* = .177, *F*(1, 255) = 54.572, *p* = < .001). It was found that higher scores on the MENQOL (indicative of poorer quality of life) significantly predicted lower scores on the BAS‐2 (*β* = −.421, *p* = < .001), indicative of lower levels of body appreciation.

## 4 DISCUSSION

In our survey of 255 women, just under half reached the EAT‐26 cut‐off raw score indicating high‐risk of an eating disorder (Baker & Runfola, 2016; Rubinstein & Foster, 2013). Participants in the high‐risk group scored significantly greater on the MENQOL and BSQ‐16, and significantly lower on BAS‐2. This suggests that women at high‐risk of an eating disorder have significantly poorer menopausal quality of life, greater preoccupation with their shape and weight, and less appreciation for their bodies compared to women at low‐risk of an eating disorder. Furthermore, when comparing specific symptoms, women in the high‐risk group scored significantly higher on the sexual, physical, and psychosocial (but not vasomotor) subscales of the MENQOL. This suggests that women at high‐risk of an eating disorder self‐report a significantly greater presence of, and are significantly more bothered by, these symptoms.

Why some women may be high‐risk is unclear. The physical, sexual, and psychosocial symptoms of the menopausal transition—such as changes in body composition, reduced libido, and loss of self‐identity—may serve to heighten the risk of eating disorders, including in those women who have no history of eating disordered cognitions or behaviors, by inducing/exacerbating body dissatisfaction and lowering self‐esteem (Mendelson et al., 2002). With the onset of these specific symptoms, women may perceive their appearances as departing from Western cultural beauty ideals of youth, thinness, and femininity (Slevec & Tiggemann, 2011). Therefore, body dissatisfaction and eating pathology may develop or intensify.

The increased susceptibility to eating disorders among women during midlife may be linked to their internalization of negative societal attitudes toward aging. Similar to the influence of the “Thin Ideal” perpetuated by Western media, which has significantly shaped body image perceptions among women globally (Witcomb et al., 2013), ageism and negative stereotypes surrounding aging may exert comparable effects on women entering midlife and the menopausal transition. Ageist stereotypes, such as the concept of “successful” aging characterized by the ability to conceal one's age and maintain a youthful appearance can induce feelings of inadequacy and body dissatisfaction among women at midlife age (Cecil et al., 2022). Interventions aimed at fostering more positive perceptions of women and aging, similar to those employed in campaigns addressing female body image (e.g., Dove campaigns), may hold promise in mitigating the adverse impact of the menopausal transition on eating and body image.

Interestingly, the severity—and negative impact—of vasomotor symptoms were not significantly different between women at high‐risk of an eating disorder and women at low‐risk. Perhaps, unlike enduring or potentially permanent psychosocial, sexual, or physical changes, vasomotor symptoms (such as hot flashes) may be perceived by women as a temporary consequence of the menopausal transition that will subside post‐ menopause (Avis et al., 2015). As a result, women may be more accepting of vasomotor symptoms; thus they are less likely to induce or exacerbate eating disorder pathology. Alternatively, this finding may be attributable to different levels of awareness regarding various menopausal symptoms. Historically, hot flashes have received more attention and documentation as a prevalent and anticipated symptom of the menopausal transition compared to other menopausal‐induced changes (Sydora et al., 2021). The normalization of the experience of these symptoms may mean that women are better equipped to anticipate and cope with these compared to other symptoms such as anxiety, weight gain, or changes in libido. As a result, the experience of severe vasomotor symptoms may be less likely to serve as triggers for the onset of body dissatisfaction or eating pathology compared to psychosocial, sexual, or physical symptoms. This could explain the observed non‐significant difference in vasomotor symptoms between women at high versus low‐risk of an eating disorder. Educational interventions targeting societal awareness of the diverse range of physical, sexual, and psychological symptoms of the menopausal transition may effectively normalize these experiences. By broadening the understanding of menopausal experiences beyond vasomotor symptoms and highlighting the potential for other manifestations associated with eating disorder risk (physical, sexual, and psychosocial), as well as supporting women with managing these changes, such interventions could potentially prevent the development of body image and eating disorder pathology during this phase of life.

Women who are at a high‐risk of developing an eating disorder not only showed more severe physical, sexual, and psychosocial symptoms, but they also appear to have significantly less appreciation for their body's capabilities and functions outside of appearance. Results from the regression analyses revealed that menopausal quality of life was a significant predictor of body appreciation, suggesting that severe and bothersome menopausal symptoms can trigger women to have less respect for, and more negative attitudes toward, their own body. This is perhaps unsurprising. However, this again may be related to women's level of education regarding the menopause and their expectations surrounding which menopausal symptoms they anticipate experiencing during the menopausal transition. These findings regarding the relationship between body appreciation and eating disorder risk are consistent with previous research on body appreciation in clinical samples, showing that higher body appreciation is associated with a reduced risk of developing eating disorder symptoms over time (Koller et al., 2020). Meta‐analyses have also confirmed the inverse relationship between body appreciation and various risk factors for eating disorders, such as eating restraint, body image dissatisfaction, and psychological distress (Linardon et al., 2022). These patterns have also been replicated in perimenopausal samples, where body appreciation was a moderator of dietary restraint (Thompson & Bardone‐Cone, 2019). It seems that interventions targeting the enhancement of body appreciation, rather than those solely reducing body dissatisfaction, may produce the most significant reductions in eating disorder symptom severity within this demographic. While research on the practical implementation of body appreciation interventions is still emerging, recent findings indicate that cultivating gratitude could serve as a strategy to enhance body appreciation (Homan & Tylka, 2018). Practical approaches aimed at redirecting women's focus toward the functionality and accomplishments of their bodies throughout their lifetime, such as childbirth or athletic achievements, rather than solely emphasizing appearance‐related aspects, may also prove beneficial in fostering body appreciation (Linardon et al., 2023).

Ultimately, body image perception is a serious and significant issue. The changes in physical appearance and function that some women experience during the menopausal transition, such as weight and shape change, sleep disruption, night sweats, and signs of ageing, all have the potential for reducing body satisfaction and body esteem (Monteleone et al., 2018; Pearce et al., 2014). Some studies also indicate that body image may have predictive value for the severity of menopausal symptoms experienced by women during the menopausal transition. Cross‐sectional analyses using the SWAN cohort (a longitudinal study of US women at midlife age which began in 1994) found those with increased body image dissatisfaction or who perceived themselves as “unattractive” had higher scores of depressive symptoms during the menopausal transition (Jackson et al., 2014). In addition, a study of 270 midlife women in the United Kingdom identified a positive association between rating highly on body dissatisfaction scales and holding negative attitudes toward menopause (Rubinstein & Foster, 2013). It appears that the onset of menopausal symptoms not only impacts body image, but women's body image can predict their menopausal quality of life. The findings of these studies highlight the complex and bidirectional nature of the relationship between body image and menopausal quality of life, and the importance of considering women's unique body image perceptions when implementing support during the menopausal transition.

### 4.1 Strengths and limitations

Certain aspects of the study require attention concerning the limitations of the findings. First, the current investigation lacks consideration of hormone‐replacement therapy (HRT) in the examination of the association between menopausal quality of life and eating disorder risk. This exclusion is notable given that HRT is shown to be effective in relieving the adverse effects of menopausal symptoms for some women (e.g., hot flashes, genitourinary symptoms (Comparetto & Borruto, 2023)) thus potentially preventing a deterioration in their quality of life. HRT could potentially reduce the likelihood of the development and exacerbation of eating and body image pathology. Future research exploring the interplay between menopausal symptoms and disordered eating behaviors, as well as body image concerns, should consider the potential impact of HRT on this relationship.

Second, despite recruitment efforts to ensure inclusivity of survey responses, and thus representation of menopausal experiences, Black, Asian, and Minority Ethnic populations and non‐binary individuals are underrepresented in the responses, with the majority of respondents being White and cisgender. Given the highly personal and diverse nature of the menopausal experience, this lack of diversity constrains a comprehensive understanding of the association between menopausal quality of life with disordered eating behaviors across various ethnic and gender identity cohorts, as well as suggestions surrounding the optimization of treatment interventions.

Finally, there are additional factors of menopausal eating disorders that require further understanding. Given the correlational nature of the study design, the results do not provide clarity on whether engaging in eating disordered cognitions and behaviors causes a decline in menopausal quality of life or if women experiencing more severe menopausal symptoms are more vulnerable to the new onset, exacerbation, or relapse of certain eating disorder behaviors or risks. Future research should consider implementing mediational analytical methods to establish the sequential mechanisms surrounding menopausal eating disorders. Furthermore, the cross‐sectional nature of the study also prevents insights into changes in menopausal quality of life and body image over time. Future longitudinal studies investigating changes in eating and body image pathology and menopausal symptomology across midlife are imperative if we are to develop a fully comprehensive understanding of, and protect against, negative effects of mid‐life changes

### 4.2 Future directions and conclusions

Clinicians and researchers evaluating women with eating disorders at midlife age should consider the potential influence of menopausal quality of life in relation to eating disorder etiology, symptom maintenance, and exacerbation. Interventions aimed at not only reducing body dissatisfaction, but those that actively bolster body appreciation may reduce eating disorders' symptom severity more effectively in this population. Interventions may be more effective if they support women with managing the sexual, physical, and psychosocial aspects of the menopausal transition to improve overall menopausal quality of life, thus treating and preventing the exacerbation of eating and body image pathology. Due to the complex, multidirectional nature of this relationship, it may be useful for future research to explore the sequential mechanisms surrounding the manifestation of eating and body image pathology during the menopausal transition to develop a fully comprehensive understanding of menopausal eating disorders. Future longitudinal studies would also provide valuable insights into how women's eating and body image pathology evolve over time, spanning midlife and the menopausal transition. Such investigations can contribute to a deeper understanding of the specific time periods during which women may be particularly vulnerable to these issues.

## AUTHOR CONTRIBUTIONS

**Sophie Temple**: Conceptualization; methodology; validation; formal analysis; investigation; writing—original draft; visualization. **Eef Hogervorst**: Conceptualization; methodology; validation; resources; writing—review and editing; supervision. **Gemma Witcomb**: Methodology; validation; formal analysis; resources; writing—review and editing; visualization; supervision.

## CONFLICT OF INTEREST STATEMENT

The authors declare no conflicts of interest.

## FUNDING STATEMENT

This research received no specific grant from any funding agency in the public, commercial, or not‐for‐profit sectors.

### PEER REVIEW

The peer review history for this article is available at https://publons.com/publon/10.1002/brb3.3609

## Data Availability

The data that support the findings of this study are available from the corresponding author upon reasonable request.
